# Complete Genome Sequence of a Divergent Isolate of Cherry Virus A from Prunus avium in China

**DOI:** 10.1128/MRA.01218-18

**Published:** 2018-10-25

**Authors:** Yunxiao Xu, Rui Gao, Shifang Li, Meiguang Lu

**Affiliations:** a State Key Laboratory for Biology of Plant Diseases and Insect Pests, Institute of Plant Protection, Chinese Academy of Agricultural Sciences, Beijing, China; Indiana University Bloomington

## Abstract

Here, we report the complete genome sequence of a divergent cherry virus A (CVA) isolate (ChYT56) from Prunus avium in China. The genome nucleotide sequence has low identity (80.7%) with a CVA from P. avium (GenBank accession number FN691959) and high identity (97%) with a CVA from P. armeniaca (GenBank accession number LC125634).

## ANNOUNCEMENT

Cherry virus A (CVA) is a member of the genus Capillovirus, family Betaflexiviridae, which was first described in Germany from sweet cherry (Prunus avium) (1). CVA has been detected in Prunus hosts, such as cherry (2), apricot, peach (3), mume, and plum (4, 5). The virus is widely distributed in more than 10 countries, including Germany (1), Canada (3), India (6), and other countries (7). Previously, we reported the complete nucleotide sequences of three Chinese isolates (ChTA11, ChTA12, and ChYT52) of CVA from cherry (7, 8). The sequences of isolates ChTA11 and ChTA12 are closely related and cluster in phylogroup I with a cherry isolate (GenBank accession number X82547). The majority of noncherry isolates cluster in phylogroup III, while isolate ChYT52, as a consequence of a recombination event that occurred between CVA isolates from cherry and noncherry hosts, clusters in phylogroup II (7, 8). However, the genetic diversity analyses among the CVA sequences derived from 31 samples in 3 genomic regions that correspond to the coat protein (CP), the RNA-dependent RNA polymerase (RdRp), and the movement protein (MP) showed that the ChYT56 cherry tree is the only sample for which only a single haplotype was detected for all 3 genes (7, 8).

The complete genome sequence was determined for a CVA isolate from a ChYT56 sweet cherry tree in Shandong Province, China. Total RNA was extracted from this ChYT56 tree isolate, and reverse transcription-PCR (RT-PCR) was performed as described by Gao et al. (7). Four pairs of PCR primers (7) and one pair of primers, F (5′-TTCCCTGACAAATCCAAAGG-3′) and R (5′-CAGTTTGGCCAAGGATGACT-3′), which direct the amplification of overlapping fragments that span the entire CVA genome, were used. The 3′ and 5′-terminal regions were amplified using an oligo(dT) primer and a 5′-full rapid amplification of cDNA ends (RACE) kit with tobacco acid pyrophosphatase (TAP; TaKaRa, Beijing, China). All amplification products were cloned and sequenced (using the ABI Prism 3730XL DNA analyzer). The resulting overlapping sequences were then assembled (using DNAMAN 6.0) into the complete genome sequence of the CVA isolate, named ChYT56.

The complete genome of isolate ChYT56 consists of 7,433 nucleotides, encodes 2 open reading frames (ORFs), and has a GC content of 39.45%. ORF1 (nucleotide positions 107 to 7135) encodes the RdRp and CP proteins, and the overlapping ORF2 (nucleotide positions 5452 to 6843) encodes the MP. The genome nucleotide sequence had low identity (80.7%) with a CVA from P. avium (GenBank accession number FN691959) and high identity (97%) with a CVA from P. armeniaca (GenBank accession number LC125634). Phylogenetic analysis of the ChYT56 genome with CVA genomes present in GenBank resulted in the same clusters as those shown by Gao et al. (7), and isolate ChYT56 clusters into phylogroup III, the cluster containing the majority of noncherry isolates. It has been suggested that ChYT56 is a highly divergent isolate from P. avium. We also found that the sequences from a few isolates from P. avium obtained using next-generation sequencing (9) clustered into phylogroup III, while ChYT56 is the only isolate sequence divided into phylogroup III from P. avium that was amplified by conventional RT-PCR.

### Data availability.

The complete genome sequence of ChYT56 has been deposited in GenBank under the accession number MH806869.
